# Workplace Bullying and Violence on Burnout Among Bangladeshi Registered Nurses: A Survey Following a Year of the COVID-19 Pandemic

**DOI:** 10.3389/ijph.2022.1604769

**Published:** 2022-10-17

**Authors:** Saifur Rahman Chowdhury, Humayun Kabir, Mahmudur Rahman Chowdhury, Ahmed Hossain

**Affiliations:** ^1^ Department of Public Health, School of Health and Life Sciences, North South University, Dhaka, Bangladesh; ^2^ Begum Rabeya Khatun Chowdhury Nursing College, Shahjalal University of Science and Technology, Sylhet, Bangladesh; ^3^ Health Services Administration, College of Health Sciences, University of Sharjah, Sharjah, United Arab Emirates; ^4^ NSU Global Health Institute (NGHI), North South University, Dhaka, Bangladesh

**Keywords:** burnout, COVID-19, nurses, Bangladesh, workplace violence, workplace bullying

## Abstract

**Objectives:** To investigate burnout among Bangladeshi nurses and the factors that influence it, particularly the association of workplace bullying (WPB) and workplace violence (WPV) with burnout.

**Methods:** This cross-sectional study collected data from 1,264 Bangladeshi nurses. Mixed-effects Poisson regression models were fitted to find the adjusted association between WPB, WPV, and burnout.

**Results:** Burnout was found to be prevalent in 54.19% of 1,264 nurses. 61.79% of nurses reported that they had been bullied, and 16.3% of nurses reported experience of “intermediate and high” levels of workplace violence in the previous year. Nurses who were exposed to “high risk bullying” (RR = 2.29, CI: 1.53–3.41) and “targeted bullying” (RR = 4.86, CI: 3.32–7.11) had a higher risk of burnout than those who were not. Similarly, WPV exposed groups at “intermediate and high” levels had a higher risk of burnout (RR = 3.65, CI: 2.40–5.56) than WPV non-exposed groups.

**Conclusion:** Nurses’ burnout could be decreased if issues like violence and bullying were addressed in the workplace. Hospital administrators, policymakers, and the government must all promote and implement an acceptable working environment.

## Introduction

Healthcare workers (HCWs) are integral parts of the health system of any country. Among them, nurses are identified as the most responsible for the patients’ better prognosis. However, working place, in some cases, becomes an issue of their adverse mental health outcomes. Previously in several studies, workplace bullying (WPB) and workplace violence (WPV) were addressed to predict nurses’ burnout [1–5]. On the other hand, burnout always predicts adverse mental health outcomes [6]. Therefore, the nurses being fit both physically and mentally were encountered in numerous research [3, 7].

Burnout is the prolonged response due to long-standing interpersonal stressors. In the 1970s, this term was introduced by psychoanalyst Freudenberger [8], and this stress syndrome has subsequently been defined by Maslach as consisting of three qualitative dimensions, which are cynicism, emotional exhaustion, and depersonalization that reduce the professional proficiency and personal accomplishment [9]. Suffering from burnout leads to less motivation which results in lower cognitive functions due to emotional exhaustion. Among health care workers, nurses are known to experience the symptoms of burnout more than others do, and this poses serious consequences for patients, other healthcare professionals, and healthcare institutions [10]. Nurses’ burnout is influenced by a multitude of factors such as work at night shifts, work-related stress, the number of days off, disagreements with co-workers or patients, as well as the connection between the nurse and their supervisor [11–13].

Workplace bullying (WPB) is defined as a pattern of offensive behavior by members of an organization, which often exacerbates in intensity with the endeavor to harm [14]. Employees perceive WPB when they become targeted and exposed to prolonged negative behaviors and cannot defend themselves [15]. WPB is significantly correlated with physical and emotional fatigue and is known as a tolerated issue in nursing [16]. Workplace bullying has a variety of negative consequences, ranging from low self-esteem to suicide [17, 18]. Furthermore, research found that several job-related problems such as lower job satisfaction, lower productivity, poor job performance, burnout, and an increased likelihood of employee turnover intent might be caused by workplace bullying [3]. Several studies investigated the occurrence of bullying and its potential consequences, particularly the relationship between workplace bullying and symptoms of burnout. The results indicated that burnout symptoms (emotional exhaustion and depersonalization) were more common and higher among nurses who reported being bullied [6, 19].

Workplace violence (WPV) is defined as the use of force against another person or a group of individuals in the workplace that causes physical or psychological harm or even death [20]. WPV is the violent act such as physical or verbal assaults and threats of assault organized by someone at the workplace. It can be perpetrated by patients, families, and co-workers. The prevalence of WPV among healthcare workers is significantly high in Asian countries: 51% in Pakistan [21], 62% in China [22], and 63% in India [23]. Besides, it is not a new issue in Bangladesh that HCWs are subjected to WPB and WPV. A recent study carried out in Bangladesh reported that a high proportion of healthcare professionals (43%) experienced some form of WPV [24]. Several studies in many countries found a relationship between WPV and burnout among nurses [25–27]. Burnout is one of the mechanisms through which WPV may result in poor psychological and physical health outcomes among nurses.

In the aftermath of the COVID-19 pandemic, numerous incidents of violence, harassment, and stigmatization have been reported against healthcare employees, patients, and medical infrastructure; 67% of the reported incidents of violence and harassment were aimed at healthcare personnel [28]. These violent actions have been found to elevate stress levels and, as a result, intensify the psychological consequences resulting from moral injuries [29]. Besides, during COVID-19, a large number of HCWs experienced unfavorable mental health outcomes. Studies have demonstrated that nurses are more prone than doctors and other healthcare workers to WPV and WPB [30, 31]. A recent study conducted in Bangladesh among nurses to determine the mental health consequences during the COVID-19 pandemic reported that the prevalence of mild to extremely severe depression was 50.5%, anxiety was 51.8%, and stress was 41.7% [32].

The majority of reports on WPV, WPB, and healthcare personnel have appeared in the media through newspapers and electronic media in Bangladesh; however, there has not been any systematic research conducted to determine the true prevalence of WPV and WPB or the impacts that they have [33]. Moreover, while it is clear that HCWs experienced a higher level of bullying and violence during COVID-19 around the world, there are no substantial evidence-based findings on these workplace hazards and their impact on burnout among Bangladeshi nurses. Henceforth, we sought to examine the burnout among the nurses of Bangladesh and its predictive factors, in particular the association of workplace bullying and violence with burnout. This finding could serve as foundational information for establishing a healthy organizational culture within the nursing community, reducing the risk of burnout among clinical nurses.

## Methods

### Study Design and Settings

This was a cross-sectional study conducted among Bangladeshi registered nurses available on online platforms and the nurses working in eight tertiary level hospitals of two large administrative divisions (Dhaka and Sylhet) in Bangladesh. These two administrative divisions were conveniently selected because Dhaka is the capital and Sylhet is a significant region of Bangladesh. The self-reported data were collected between 26 February 2021 and 10 July 2021.

### Participants

The study population of this study was all registered nurses of Bangladesh working in clinical settings for at least 1 year. Our required sample size was 1,024 at 80% power, 95% CI of 0.05 to 1.96, and 3% margin of error with an assumption that 50% of the nurses had symptoms of burnout. Finally, from 1,345 obtained responses, 1,264 completed responses were considered in the analysis. Thus, an additional 11% of participants (240) helped to reduce the study’s margin of error [34].

### Data Collection Procedure

An online and offline method of data collection was approached in this study. A semi-structured self-response questionnaire was developed to gather data for this study’s findings. The questionnaire had five parts. On the front page, study’s objectives and the responding procedure were described. The first part of the questionnaire consisted of the demographic and occupational characteristics of the respondents. In the subsequent parts of the questionnaire, workplace bullying, violence, and burnout-related questions were documented with proper instructions for responding to them. Finally, three dichotomous-response (yes/no) questions related to participants’ “presence of enjoyment in the current job,” “presence of enthusiasm in the current job,” and “presence of satisfaction in the current job” were asked. The questionnaire was translated into Bangla with the help of an expert. One nurse superintendent of a tertiary hospital and one public health expert in Bangladesh reviewed the initial questionnaire. Based on their suggestions, modification of the questionnaire was performed. For online data collection, convenience and snowball-sampling methods were followed. At the time of the pandemic, face-to-face data collection was restricted. So, a questionnaire link (using “Google Form”) was distributed on common social media platforms used by nurses (Facebook, WhatsApp, etc.) in Bangladesh, and available registered nurses were invited to participate. By online method of data collection, 721 completed responses were obtained. To achieve the required sample size, we distributed another 700 printed questionnaires in eight hospitals in two geographical divisions (Dhaka and Sylhet) in Bangladesh. The respondents were provided with 7 days to respond to the questionnaire, and data collectors received questionnaires after 7 days. After receiving 655 returned copies, 543 were obtained as completed responses. Thus, a total of 1,264 completed responses were finally included in the current study.

### Workplace Bullying Measure

The Short Negative Acts Questionnaire [S-NAQ] was used to determine workplace bullying exposure [35]. It comprises nine items that assess whether or not a person has been subjected to bullying behaviors in the last 6 months. The scale items address both the personal and work-related forms of bullying (e.g., “there has been gossip or rumors spread about you” and “necessary information was withheld that impeded your ability to do your job”). The answer categories ranged from 1 to 5, with 1 being “never” and 5 being “daily”. The S-NAQ has a good level of reliability and validity [35]. This scale has been used to measure bullying in numerous studies in several countries, including Belgium, Italy, and Norway [36–38]. The 9-item S-NAQ has a Cronbach's α of 0.89 in our current study. S-NAQ has two cut-off scores: 15 and 23, which means that respondents scoring less than 15 in the S-NAQ can be considered “non-exposed” to bullying at work; those scoring between 15 and 22 are at “high risk” of being bullying victims or may be immersed in a bullying process; whereas those scoring 23 or higher can be considered “targeted” of workplace bullying [39].

### Workplace Violence Measure

The workplace violence scale (WVS) was used to measure the WPV experienced in the last 12 months [40, 41]. Several studies used this scale to measure workplace violence among nurses and other health care workers previously [42–45]. The scale was composed of five types of WPV named physical assault, emotional abuse, threat, verbal and sexual harassment, and sexual assault. The responses (score ranges from 0 to 3) of the five items scale indicate the frequencies of each type of WPV. Score 0 indicates WPV 0 time/none, 1 indicates 1 time, 2 indicates 2 or 3 times, and 3 indicates WPV more than 4 times. By summing all the responses, the total score ranged from 0 to 15. Four WPV categories were derived from scores 0 for none, 1 to 5 for low, 6 to 10 for intermediate, and 11 to 15 for high. In the questionnaire, participants were given specific definitions of each type of violence. Cronbach’s α reliability coefficient was calculated at 0.60, which indicated an acceptable internal consistency of the scale.

### Burnout Measure

The 10-item Burnout Measure-Short version (BMS) developed by Malach-Pines was used in the last segment of our survey [46]. The BMS is a brief and easy-to-use tool to measure burnout. In this tool, an individual’s levels of physical exhaustion, emotional exhaustion, and mental exhaustion are assessed using the core elements of the concept of burnout in a series of 10 questions. Each item is graded on a 7-point Likert scale from 1 (never) to 7 (always). For all 10 items, the total response points ranged from 10 to 70, based on the response value for each item. The overall burnout score for each participant was calculated by dividing the sum of each participant’s response values by 10. Thus, the overall burnout score ranged from 1 to 7. An overall score ≥4 indicates an established state of burnout, according to Malach-Pines. Therefore, individuals are divided into two categories based on their overall burnout score: those who are likely to experience burnout (overall score ≥4) and those who are not likely to experience burnout (overall score <4). The 10-item BMS was validated and used on different samples and has shown satisfactory psychometric properties [46–50]. In our study, the Cronbach’s α for the 10-item BMS was 0.89, indicating good reliability.

### Data Analysis

The demographic profile and occupational characteristics of the study sample were described using descriptive statistics expressed in frequency and percentages. A chi-square test was performed for unadjusted associations. Mixed-effects Poisson regression models were fitted to find the adjusted association between burnout and WPB, WPV, and other study variables. The factors in the unadjusted test were included in the adjusted models at *a priori* specified *p*-value of 0.1. Mixed-effects Poisson regression models with robust error variance were used to avoid overestimation of associations with common binary outcomes measured in cross-sectional studies [51–53]. We fitted three models to investigate the adjusted association between the predictor variables and the outcome variable. Model 1 included only demographic variables. Model 2 included both demographic and occupational variables. Model 3 included all main-effects terms (demographic and occupational variables) and two-way interaction-effects terms between WPB and WPV. The findings were expressed as relative risks (RRs) with 95% confidence intervals (CIs). Moreover, the association between burnout and three job-related questions was evaluated using chi-square test. All statistical analyses were two-sided, and a *p*-value < 0.05 was considered statistically significant. Statistical software STATA-16 (Stata Corp LP, College Station, TX, United States) was used for data analysis.

### Ethical Issue

The ethical review committee of Begum Rabeya Khatun Chowdhury Nursing College, Bangladesh (approval ID: BRKCNC-IRB-2021/5) approved this study involving human participants. On the first page of the questionnaire, the study’s aims and objectives were explained. The participation of respondents in the study defined their implied consent.

## Results

### Characteristics of the Participants

The characteristics of the 1,264 participants are presented in Table 1. The mean age of the participants was 28.41 (SD: 5.54), and 70.02% were female. The 756 (59.81%) nurses were involved in government hospitals, and the remaining 508 (40.19%) were in private hospitals. Moreover, 910 (71.99%) of the nurses were from tertiary care hospitals. More than half of the nurses (52.93%) reported that they did not have enough equipment to manage the patients while on the job. A substantial percentage of respondents (79.27%) stated that they had never been awarded for good work, and 81.80% of nurses stated that they had never been trained against WPV.

**TABLE 1:** Characteristics of the participants, Bangladesh, 2021 (*n* = 1,264).

Variables	*n*	Percent/mean (SD)
Demographic variables		
Mean age, years	1,264	28.41 (5.54)
Age group, years		
<25	303	23.97
25–29	604	47.78
≥30	357	28.24
Sex		
Male	379	29.98
Female	885	70.02
Geographical division of workplace		
Dhaka	618	48.89
Chattagram	132	10.44
Sylhet	375	29.67
Others^a^	139	11.0
Educational degree		
Masters or above	230	18.20
Bachelor	514	40.66
Diploma	520	41.14
Marital status		
Married	675	53.40
Unmarried	589	46.60
Occupational variables		
Type of job		
Government	756	59.81
Private	508	40.19
Level of hospital		
Tertiary	910	71.99
Secondary	207	16.38
Primary	147	11.63
Monthly income		
<20,000 BDT	282	22.31
20,000–30,000 BDT	592	46.84
>30,000 BDT	390	30.85
Job experience		
<3 years	498	39.40
≥3 years	766	60.60
Got salary timely		
Yes	1134	89.72
No	130	10.28
Working hours (*n* = 1,261)		
≤36 h	597	47.34
37–48 h	520	41.24
>48 h	144	11.42
Daily sleeping hours		
<8 h	803	63.53
≥8 h	461	36.47
Average patients served per day (*n* = 1,262)		
<20	329	26.07
20–39	449	35.58
≥40	484	38.35
Had sufficient equipment to manage patients		
Yes	595	47.07
No	669	52.93
Had rewards for good job performance		
Yes	262	20.73
No	1,002	79.27
Had training against WPV		
Yes	230	18.20
No	1,034	81.80
WPB		
Non-exposed	483	38.21
High risk	529	41.85
Targeted	252	19.94
WPV		
None	328	25.95
Low	730	57.75
Intermediate and high	206	16.30
Burnout		
Yes	685	54.19
No	579	45.81

SD, standard deviation.

n, number.

WPV, workplace violence.

WPB, workplace bullying.

^a^ Others = Rajshahi, Khulna, Barishal, Rangpur, Mymensingh.

### Prevalence of Burnout, Workplace Bullying, and Workplace Violence

Burnout was found to be prevalent in 685 (54.19%) of the 1264 nurses. Among the nurses, 781 (61.79%) reported workplace bullying (WPB), with 529 (41.85%) and 252 (19.94%) nurses reported “high risk bullying” and “targeted bullying,” respectively. According to the survey, 206 nurses (16.30%) reported “intermediate and high” levels of workplace violence during the last 12 months (Table 1).

### Unadjusted Association of Workplace Bullying and Workplace Violence with Burnout

Table 2 represents the burnout distribution as well as the unadjusted relationship between burnout and WPB, WPV, and other factors. In the chi-square test, WPB was found to be significantly associated with burnout of nurses (*p* < 0.001). Similarly, WPV was significantly associated with nurses’ burnout (*p* < 0.001). Almost half of the female nurses (49.27%) were found to be burnout. In demographic variables, sex, geographical division of workplace, and educational degree of the nurses were found to be significantly associated with burnout (*p* < 0.001). The occupational variables that were significantly associated with burnout were job experience of the nurses, timely salary, working hours, sleeping hours, number of patients dealt with per day, the sufficiency of the equipment to manage patients, rewards for good job performance, and training for the nurses against WPV (*p* < 0.001).

**TABLE 2:** Unadjusted association between burnout and workplace bullying, violence, and other study variables, Bangladesh, 2021 (*n* = 1,264).

Variables	Burnout			
	*n*	Percent (%)	χ^2^	*p*-value
Demographic variables				
Age, years				
<25	148	48.84	1.55	0.462
25–29	269	44.54		
≥30	161	45.38		
Sex				
Male	143	37.73	14.22	**<0.001**
Female	436	49.27		
Geographical division of workplace				
Dhaka	310	50.16	16.779	**0.001**
Chattagram	63	47.73		
Sylhet	139	37.07		
Others^a^	67	48.20		
Educational degree				
Masters or above	117	50.87	8.14	**0.017**
Bachelor	248	48.25		
Diploma	214	41.15		
Marital status				
Married	306	45.33	0.13	0.172
Unmarried	273	46.35		
Occupational variables				
Type of job				
Government	348	46.03	0.04	0.845
Private	231	45.47		
Level of hospital				
Tertiary	411	45.16	0.54	0.762
Secondary	98	47.34		
Primary	70	47.62		
Monthly income				
<20,000 BDT	134	47.52	0.99	0.609
20,000–30,000 BDT	274	46.28		
>30,000 BDT	171	43.85		
Job experience				
<3 years	246	49.40	4.27	**0.039**
≥3 years	333	43.47		
Got salary timely				
Yes	493	43.47	24.17	**<0.001**
No	86	66.15		
Working hours (*n* = 1,261)				
≤36 h	277	46.40	13.69	**0.001**
37–48 h	217	41.73		
>48 h	85	59.03		
Daily sleeping hours				
<8 h	387	48.19	5.06	**0.025**
≥8 h	192	41.65		
Average patients served per day (*n* = 1,262)				
<20	135	41.03	11.84	**0.003**
20–39	192	47.76		
≥40	251	51.86		
Had sufficient equipment to manage patients				
Yes	229	38.49	24.26	**<0.001**
No	350	52.32		
Had rewards for good job performance				
Yes	86	32.82	22.44	**<0.001**
No	493	49.20		
Had training against WPV				
Yes	74	32.17	21.05	**<0.001**
No	505	48.84		
WPB				
Non-exposed	111	22.98	240.72	**<0.001**
High risk	260	49.15		
Targeted	208	82.54		
WPV				
None	80	24.39	126.45	**<0.001**
Low	347	47.53		
Intermediate and high	152	73.79		

n, number.

WPV, workplace violence.

WPB, workplace bullyings.

^a^ Others = Rajshahi, Khulna, Barishal, Rangpur, Mymensingh.

p-values appearing in bold are statistically significant.

### Adjusted Analysis: Mixed-Effects Poisson Regression Models

Table 3 represents the adjusted association of workplace bullying, burnout, demographic, and occupational factors with burnout identified from three mixed-effects Poisson regression models. According to **Model 3**, among the nurses, “high risk bullying” and “targeted bullying” were at 2.29 (95% CI: 1.53–3.41, *p* < 0.001) and 4.86 (95% CI: 3.32–7.11, *p* < 0.001) times more risk of burnout, respectively, compared to non-exposed groups to the bullying. The “low” and “intermediate and high” levels of WPV groups were 1.48 (95% CI: 1.03–2.11, *p* < 0.033) and 3.65 (95% CI: 2.40–5.56, *p* < 0.001) times more risk of being burnout, respectively, compared to WPV non-exposed groups. In terms of their association with burnout, WPB and WPV were found to interact with each other, as two combinations of WPB and WPV were significant (high risk bullying × intermediate and high WPV, *p* = 0.002 and targeted bullying × intermediate and high WPV, *p* < 0.001) compared with their respective reference combinations. Females were at 1.35 (95% CI: 1.19–1.53, *p* < 0.001) times more risk of burnout. Compared to the nurses who completed diploma educational degrees, the nurses who completed bachelor’s and master’s degrees were at 1.21 (95% CI: 1.05–1.40, *p* = 0.010) and 1.14 (95% CI: 1.00–1.28, *p* = 0.042) times more risk of being burnout, respectively. Less experienced (<3 years) nurses were at 1.33 (95% CI: 1.19–1.49, *p* < 0.001) times more risk of being burnout compared to the nurses with ≥3 years of job experience. Nurses with higher working hours (>48 h per week) were at 1.22 (95% CI: 1.04–1.42, *p* = 0.014) times more risk of being burnout than the nurses who worked ≤36 h per week. The nurses who served the highest number of patients (≥40 patients per day) were at 1.20 (95% CI: 1.05–1.38, *p* = 0.008) times more risk of being burnout than the nurses who served <20 patients per day. The nurses who usually did not get rewards from their authority for good job performance were at 1.33 (95% CI: 1.13–1.58, *p* = 0.001) times more risk of burnout than their counterparts. Nurses who did not receive any training against WPV were at 1.21 (95% CI: 1.02–1.44, *p* = 0.031) times more risk of burnout than their opposite parts.

**TABLE 3:** Mixed-effects Poisson regression models to find the adjusted association between burnout and workplace bullying, violence, and other study variables, Bangladesh, 2021 (*n* = 1,264).

Variables	Burnout								
	Model 1			Model 2			Model 3		
	RR	95% CI	*p*-value	RR	95% CI	*p*-value	RR	95% CI	*p*-value
Demographic variables									
Sex									
Male	Reference			Reference			Reference		
Female	1.32	1.14–1.53	**<0.001**	1.34	1.18–1.52	**<0.001**	1.35	1.19–1.53	**<0.001**
Geographical division of workplace									
Dhaka	Reference			Reference			Reference		
Chattagram	1.01	0.83–1.23	0.930	0.99	0.83–1.19	0.915	0.95	0.80–1.14	0.601
Sylhet	0.76	0.65–0.88	**<0.001**	0.79	0.69–0.91	**0.001**	0.77	0.67–0.88	**<0.001**
Others^a^	0.97	0.80–1.17	0.749	0.95	0.80–1.12	0.521	0.88	0.75–1.04	0.147
Educational degree									
Masters or above	1.25	1.06–1.48	**0.007**	1.22	1.05–1.42	**0.008**	1.21	1.05–1.40	**0.010**
Bachelor	1.19	1.04–1.36	**0.012**	1.14	1.01–1.29	**0.039**	1.14	1.00–1.28	**0.042**
Diploma	Reference			Reference			Reference		
Occupational variables									
Job experience									
<3 years				1.31	1.17–1.47	**<0.001**	1.33	1.19–1.49	**<0.001**
≥3 years				Reference			Reference		
Got salary timely									
Yes				Reference			Reference		
No				1.12	0.97–1.30	0.136	1.13	0.97–1.31	0.110
Working hours (*n* = 1261)									
≤36 h				Reference			Reference		
37–48 h				0.93	0.82–1.04	0.234	0.93	0.82–1.05	0.240
>48 h				1.21	1.03–1.42	**0.019**	1.22	1.04–1.42	**0.014**
Daily sleeping hours									
<8 h				Reference			Reference		
≥8 h				0.93	0.83–1.05	0.235	0.93	0.83–1.04	0.222
Average patients served per day (*n* = 1262)									
<20				Reference			Reference		
20–39				1.05	0.90–1.22	0.543	1.05	0.91–1.22	0.503
≥40				1.21	1.06–1.39	**0.006**	1.20	1.05–1.38	**0.008**
Had sufficient equipment to manage patients									
Yes				Reference			Reference		
No				1.04	0.91–1.17	0.585	1.03	0.91–1.17	0.614
Had rewards for good job performance									
Yes				Reference			Reference		
No				1.33	1.12–1.56	**0.001**	1.33	1.13–1.58	**0.001**
Had training against WPV									
Yes				Reference			Reference		
No				1.21	1.02–1.44	**<0.001**	1.21	1.02–1.44	**0.031**
WPB									
Non-exposed				Reference			Reference		
High risk				1.88	1.56–2.27	**<0.001**	2.29	1.53–3.41	**<0.001**
Targeted				2.83	2.34–3.42	**<0.001**	4.86	3.32–7.11	**<0.001**
WPV									
None				Reference			Reference		
Low				1.33	1.09–1.63	**0.005**	1.48	1.03–2.11	**0.033**
Intermediate and high				1.56	1.29–1.94	**<0.001**	3.65	2.40–5.56	**<0.001**
Interaction effects									
WPB × WPV									
High risk bullying × Low WPV							0.83	0.53–1.33	0.444
High risk bullying × Intermediate and high WPV							0.44	0.26–0.74	**0.002**
Targeted bullying × Low WPV							0.65	0.42–1.01	0.057
Targeted bullying × Intermediate and high WPV							0.25	0.15–0.40	**<0.001**

n, number.

RR, relative risk.

WPV, workplace violence.

WPB, workplace bullying.

Model 1 included only demographic variables.

Model 2 included demographic and occupational variables.

Model 3 included all main-effects terms (demographic and occupational variables) and two-way interaction-effects terms between WPB, and WPV.

^a^ Others = Rajshahi, Khulna, Barishal, Rangpur, Mymensingh.

p-values appearing in bold are statistically significant.

### Association Between Burnout and Three Job-Related Questions

We asked nurses three job-related questions. Compared to the nurses who suffered burnout, non-burnout nurses found enjoyment in job (46.18% vs 69.46%, *p* < 0.001), were enthusiastic (44.02% vs 67.25%, *p* < 0.001), and satisfied (42.11% vs. 65.35%, *p* < 0.001) with their present job (Table 4).

**TABLE 4:** Association between burnout and three job-related questions, Bangladesh, 2021 (*n* = 1,264).

Burnout	I Find real enjoyment in my job (*n* = 1,257)				I am enthusiastic about my job (*n* = 1,258)				I Feel satisfied with my job (*n* = 1,258)			
	n (Yes)	%	χ^2^	*p*-value	N (Yes)	%	χ^2^	*p*-value	n (Yes)	%	χ^2^	*p*-value
Yes	266	46.18	69.78	**<0.001**	254	44.02	68.64	**<0.001**	243	42.11	68.02	**<0.001**
No	473	69.46			458	67.25			445	65.35		

p-values appearing in bold are statistically significant.

## Discussion

The current study investigated burnout among Bangladeshi nurses for the first time and its associated factors, including the roles of WPB and WPV on burnout. Several factors may influence healthcare workers’ physical, mental, and social health status in a lower-middle-income country.

This study found a high prevalence of burnout (54.19%) among Bangladeshi nurses. Previous research conducted in different settings found different degrees of burnout among the nurses. For instance, burnout was found among nurses in China at 25.01% [1], in Australia at 67% [2], and in Nigeria at 76% [54], measured by the same tool used in the current study. Our study suggests that the bullied nurses were at higher risk of burnout. Numerous studies reported that WPB was significantly associated with nurses’ burnout, supporting current study findings [3, 4, 6]. This study found that WPV-exposed nurses were at a greater risk of burnout. Similarly, Liu et al. [1], Duan et al. [5], and Hamdam et al. [55] reported a positive association between WPV and burnout. However, burnout may affect nurses’ mental health and elevate turnover intention, impeding better patient care [3]. Having had no training against WPV was also associated with nurses’ burnout. Therefore, appropriate managerial support, training, and leadership with fair and equitable distribution of facilities are needed to reduce violence and bullying in the workplace.

We did not find any significant association between the nurses’ age and burnout. This finding is supported by Liu et al.’s study finding [56]. However, Hayes et al. reported that the nurses of higher age with more working experience had a lower level of burnout [57]. We found that female nurses were more prone to burnout. Female nurses’ emotional labor as a caregiver may be a reason to perceive higher burnout than men [58]. However, few studies conducted in developed countries found no gender-based variations [3, 56]. Therefore, further gender perspective factors investigation is essential in the context of Bangladesh. Nurses from the Sylhet division, the northeastern part of Bangladesh, were more affected by burnout. Consistent with our findings, Hamdan et al. found burnout variations in the country’s different geographical locations [55]. In our study, the nurses who held higher educational degrees exerted a higher level of burnout. On the contrary, Zhang et al. found a negative correlation between higher degree education and burnout among Chinese nurses [59]. However, Liu et al. observed that educational degree does not arbitrate the burnout variations [56]. In Bangladesh, nursing is known to have less social recognition, payments, and work status and is recognized as a second segmental profession [60]. Our finding may be explained by the fact that nurses with high educational degrees remain more vulnerable to psychological suppression considering the social impression, minimal job promotion, or salary increments in the context of Bangladesh [32].

Among the occupational characteristics, the nurses with less duration of working experience had a higher level of burnout, supported by the finding of Kim et al. [3]. Other similar research also found a significant correlation between nurses’ years of experience and burnout [56, 61]. Evidence suggests that along with working experience increment, nurses achieve higher tolerances to overcome any adverse working situations [61]. Thus, the working experience might play a protective role against burnout. A higher working hour was associated with a higher level of nurses’ burnout. This finding is consistent with Hayes et al.’s research [57]. Similarly, Vandenbroeck et al. reported that more working hours indicate a higher workload that affects the nurses’ burnout [62]. The current study found that dealing with more patients was also associated with burnout. Evidence suggests that emotional exhaustion is emphasized by working hours or workload increments related to nurses’ insufficient professional efficacy [63]. Therefore, synergies between working hours and working load are needed to get a service-oriented nursing practice. Having had no arrangement of rewards for the nurses from the authority for good work performance was associated with a higher level of burnout. This finding might be explained by Hayes et al.’s and Vandenbroeck et al.’s study findings that reported a lack of support from co-workers and organizations strongly associated with burnout [57, 62]. Based on this study and the studies discussed, it can be concluded that to abate burnout, rewards for good job performance and keeping nurses in decision-making need to be prioritized.

In this study, the nurses who were not suffered from burnout were more satisfied, enjoyed, and enthusiastic with their present job than those who experienced burnout. Consistently, Roy et al. investigated Bangladeshi physicians’ burnout levels that significantly predicted their job satisfaction [64]. Similarly, Demerouti et al. reported that lower life satisfaction and enjoyment are strongly associated with nurses’ experiences of higher burnout [7]. The research findings expound that burnout can erase nurses’ working enjoyment, enthusiasm, and satisfaction.

### Implications of the Findings

Nurses are the most important staff in a country’s healthcare system as they deal with the health and well-being of the patients. We anticipate that our study findings could help the authorities, the Directorate General of Health Services, the Ministry of Health and Family Welfare, and the Directorate General of Nursing and Midwifery, Bangladesh to understand the workplace environment of nurses and take the necessary measures to reduce the frequency of workplace bullying and violence.

### Strengths and Limitations

To our best knowledge, this is the first study that addressed Bangladeshi nurses’ burnout status and its associated factors, including the association with WPB and WPV. Nurses from all geographical divisions of the country got opportunities to participate in the study. The larger sample size might provide substantial accuracy to the study findings. However, this research had some limitations also. As a non-random sampling technique was applied, selection bias could not be excluded. The risk of information bias might be present due to the self-reported questionnaire. As a nature of a cross-sectional study, causality could not be established. However, we hope that the outcomes of our research will provide a succinct summary of the working environment of Bangladeshi nurses. Further in-depth and rigorous research is recommended focusing on nurses’ burnout, WPB, and WPV to establish sustained evidence to make nurses’ working environments safer.

### Conclusion

A high prevalence of burnout, workplace violence, and bullying was found among Bangladeshi nurses. Workplace violence and bullying were also identified as potential predictors of burnout. Burnout is known to have a negative impact on job satisfaction, which leads to a higher likelihood of turnover in the workplace. As a result, establishing a safer working environment is critical, as is demanding improved nursing services. Thus, addressing the variables, enhancing the working environment for nurses, increasing job satisfaction, as well as lowering burnout, bullying, and workplace violence are crucial through support and the implementation of suitable policies from hospital administrators, policymakers, and the government.
